# Revisiting the critical weight hypothesis for regulation of pubertal timing in boys

**DOI:** 10.1093/ajcn/nqaa304

**Published:** 2020-11-12

**Authors:** Maria Bygdell, Jenny M Kindblom, John-Olov Jansson, Claes Ohlsson

**Affiliations:** Centre for Bone and Arthritis Research, Department of Internal Medicine and Clinical Nutrition, Institute of Medicine, Sahlgrenska Academy, University of Gothenburg, Gothenburg, Sweden; Centre for Bone and Arthritis Research, Department of Internal Medicine and Clinical Nutrition, Institute of Medicine, Sahlgrenska Academy, University of Gothenburg, Gothenburg, Sweden; Region Västra Götaland, Sahlgrenska University Hospital, Pediatric Clinical Research Center, Gothenburg, Sweden; Department of Physiology, Institute of Neuroscience and Physiology, Sahlgrenska Academy, University of Gothenburg, Gothenburg, Sweden; Centre for Bone and Arthritis Research, Department of Internal Medicine and Clinical Nutrition, Institute of Medicine, Sahlgrenska Academy, University of Gothenburg, Gothenburg, Sweden; Region Västra Götaland, Sahlgrenska University Hospital, Department of Drug Treatment, Gothenburg, Sweden

**Keywords:** cohort study, childhood BMI, childhood body weight, puberty, peak height velocity

## Abstract

**Background:**

Recent findings indicate that there is a body weight–sensing homeostatic regulation of body weight in postpubertal rodents and humans. It is possible that body weight sensing also might be involved in the regulation of pubertal timing. Although an early small study suggested that there is a critical body weight for pubertal timing in girls, most studies have focused on BMI and reported an inverse association between BMI and pubertal timing.

**Objectives:**

In the present longitudinal well-powered cohort study, we revisited the critical weight hypothesis and tested if prepubertal body weight is a more robust inverse predictor of pubertal timing than prepubertal BMI in boys.

**Method:**

We included men born during 1945–1961 (old cohort; *n* = 31,971) and men born during 1981–1996 (recent cohort; *n* = 1465) in the large BMI Epidemiology Study (BEST) Gothenburg (combined BEST cohort *n* = 33,436). Men with information on prepubertal body weight and BMI at 8 y of age and age at peak height velocity (PHV; an objective measure of pubertal timing) were included.

**Results:**

Body weight explained more of the variance in age at PHV than BMI in both the old cohort and the recent cohort (combined cohort, body weight 6.3%, BMI 3.6%). Both body weight (*β*: −0.24 SD/SD increase in weight; 95% CI: −0.25, −0.23) and BMI (*β*: −0.18 SD/SD increase in BMI, 95% CI: −0.19, −0.17) were inversely associated with age at PHV but the association for body weight was significantly more pronounced than the association for BMI (*P* < 0.001).

**Conclusions:**

In conclusion, prepubertal body weight is a more robust inverse predictor of pubertal timing than prepubertal BMI in boys. We propose that body weight sensing constitutes a feedback mechanism to regulate pubertal timing.

## Introduction

The transition from childhood to adulthood through puberty is one of the milestones in human development, and age at pubertal timing has been shown to be associated with adult health and disease. With regard to physiological effects, an early puberty will prolong the reproductive period but reduce the length of the prepubertal growth period, which, in turn, can reduce final body size (1, 2). A large UK Biobank study showed an association between early puberty and a number of adverse health outcomes, including cardiovascular disease, type 2 diabetes, and cancers (3). Moreover, we recently discovered that early pubertal timing is associated with increased risk of adult type 2 diabetes, independent of childhood BMI (kg/m^2^) (4). In contrast, late puberty was associated with increased risk of adult fractures (5).

Despite clear associations between pubertal timing and different health outcomes, the regulation of the initiation of puberty is still poorly understood. It most likely involves an activation of the gonadotropin-releasing hormone (GnRH) pulse generator, but the underlying mechanism is unclear (6–8).

Recent findings indicate that there is a body weight sensing homeostatic regulation of body weight, the gravitostat, in postpubertal rodents and humans (9–11). In addition, an early small study suggested that there is a “critical body weight” for pubertal timing in girls, originally suggesting that girls need to reach a certain level of body fat mass before puberty can be initiated (12, 13). We therefore hypothesized that body weight sensing might be involved in the regulation of pubertal timing. It is well established that BMI is inversely associated with pubertal timing in girls (14–23). However, the results for the association between BMI and pubertal timing in boys are more inconsistent. Although several studies have reported inverse associations for childhood BMI, overweight, and obesity with pubertal timing, other studies have reported no association or a direct association (14, 16, 19, 23–30). Most, but not all, association studies in both girls and boys have focused on BMI. Some of these have suggested that fat-derived leptin may mediate the association between BMI and pubertal timing (31). A few studies have indicated that both body weight and BMI are inversely associated with pubertal timing, but to our knowledge there has been no previous study directly comparing the strengths of the associations for prepubertal BMI and weight with pubertal timing in boys (8, 18, 32, 33). In the well-powered BMI Epidemiology Study (BEST) Gothenburg cohort, information on weight and BMI at prepubertal age 8 y as well as objectively determined age at peak height velocity (PHV), an objective measurement of pubertal timing, are available for boys. In the present longitudinal study, we revisited the critical weight hypothesis for regulation of pubertal timing and tested if prepubertal body weight is a more robust inverse predictor of pubertal timing than prepubertal BMI in boys in the BEST Gothenburg cohort.

## Subjects and Methods

The primary outcome variable for the study was age at PHV.

### Data collection and study population

The population-based BEST Gothenburg cohort includes individuals who completed school in Gothenburg municipality and afterward had their school health record stored in the regional archive. The school health records include data on weight and height from regular health visits at child healthcare centers and school healthcare facilities throughout childhood, until the children finished secondary school. These health visits include all children in Sweden (>98.5% of all children in the municipality for school healthcare from calendar year 1952, corresponding to birth year 1945). The present study included boys born between 1945 and 1961 (old cohort) and a younger cohort of boys born between 1981 and 1996 (recent cohort). We also collected weight and height at young adult age from mandatory military conscription tests. Conscription was mandatory until 2010 for all Swedish men. The inclusion criteria for this study were to have ≥1 measurement of childhood weight and BMI at 6.5–9.5 y of age as well as age at PHV available. Individuals were excluded if a personal identity number (PIN) was missing (**Supplementary Figure 1**). For subjects available from the Swedish national conscription register, we compared young adult body weight, height, and BMI for those included in the present study with those not included. For the old cohort, the included subjects (*n* = 29,305) had slightly higher young adult body weight (+0.4%, *P* < 0.001), height (+0.9%, *P* < 0.001), and BMI (+0.3%, *P* = 0.022 using *t*-test) than those not included (*n* = 5386). For the recent cohort, there were no significant differences in young adult body weight (+1.4%, *P* = 0.46), height (0.0%, *P* = 0.97), or BMI (+1.4%, *P* = 0.41) for the included subjects (*n* = 641) compared with those not included (*n* = 84) in the present study. The ethics committee at the University of Gothenburg, Sweden, approved the study.

### Linkage with register from Statistics Sweden

In Sweden, a PIN is assigned to every citizen at birth or immigration. The individuals’ PINs, were used to link the BEST cohort with the Longitudinal Integration Database for Health Insurance and Labour Market Studies at Statistics Sweden, and the country of birth for every study participant and their parents were retrieved.

### Anthropometric variables

As previously described, all prepubertal BMI and weight measurements available in the age interval of 6.5 to 9.5 y were used and then age-adjusted to 8 y (34, 35). In order to avoid the confounding effect of early puberty, we also performed sensitivity analyses using measurements of weight and BMI only in the age interval 6.5–8 y. To adequately calculate age at PHV in an unbiased manner, height measurements before, during, and after the pubertal period are required. We calculated age at PHV according to a modified Infancy-Childhood-Puberty model, as previously described (30, 36, 37). The model was fitted by minimizing the sum of squares by using a modification of the Levenberg-Marquardt algorithm and including tests controlling for convergence. A good model fit was further confirmed through visual inspection of all curves (MB, JMK). Age at PHV was defined as the age at maximum growth velocity during puberty and was estimated by the model. We also performed sensitivity analyses excluding individuals with age at PHV below 11.5 y and in addition performed sensitivity analyses including only individuals born in Sweden and with parents born in Sweden.

### Statistical analyses

**FIGURE 1:**
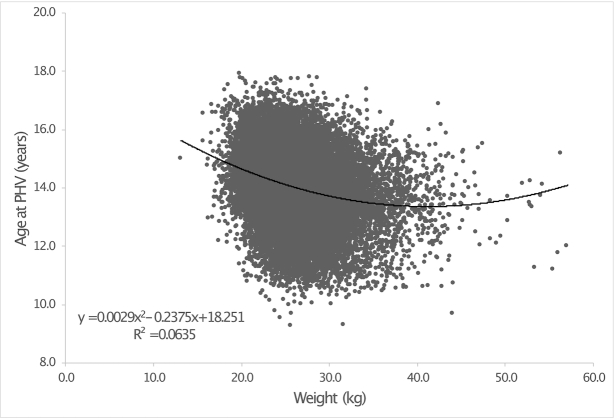
Association between weight at 8 y of age and age at PHV. Data from participants in the combined cohort (*n* = 33,436). PHV, peak height velocity.

Descriptive statistics for childhood weight, BMI, and age at PHV were calculated for the old, the recent, and the combined cohorts. The associations for childhood weight and BMI with age at PHV were evaluated using linear regression models. To facilitate the visual interpretation of the body weight finding in **Figure 1**, we presented the body weight in kilograms. The associations for both body weight and BMI in the association models in Table 3 are presented per SD increase of these parameters when used as nontransformed. As the distribution of childhood weight and BMI was slightly positively skewed, all analyses were repeated with the use of log-transformed childhood weight and BMI, with similar results. A possible nonlinear association was assessed through the inclusion of a quadratic term in the association model (a *P* value of <0.05 for the quadratic term suggested a nonlinear association) and further explored and visualized (Figure 1) using the built-in nonlinear quadratic functions in Microsoft Excel (version 16.37). The calculation of age-adjusted BMI and a *z*-test were performed in R (version 3.4.2, www.r-project.org). For all other statistical analyses, SPSS (IBM SPSS) version 26 was used.

The funding sources of the study had no role in the study design, implementation, analysis, and interpretation of the data.

## Results

Descriptive statistics for participants in the old (*n* = 31,971), the recent (*n* = 1465), and the combined (*n* = 33,436) BEST Gothenburg cohorts are presented in **Table 1**. Body weight and BMI at 8 y of age were higher and age at PHV was significantly lower in the recent cohort than in the old cohort (Table 1). We first evaluated the associations of weight and BMI at 8 y with age at PHV in unadjusted linear regression models. Both body weight and BMI were inversely associated with age at PHV. However, body weight explained substantially more of the variance in age at PHV than BMI in both the old cohort and the recent cohort (combined cohort, body weight: 5.9%; BMI: 3.4%; **Table 2**). In the combined cohort, body weight in combination with BMI did not explain more of the variance in age at PHV than only body weight (Table 2). The variance explained by body weight was slightly more pronounced in a quadratic model (combined cohort: 6.3%; Table 2, Figure 1). The shape of the association between body weight at 8 y and age at PHV revealed that the inverse association was linear in the part of the distribution in which most individuals are found, but slightly attenuated at the highest body weight end. The lowest predicted age at PHV (13.4 y) was observed for a body weight of 40.9 kg at 8 y (corresponding to +2.2 *z*-scores when using the CDC growth charts for 8-y-old boys as the reference. [(38); Figure 1].

**TABLE 1:** Descriptive statistics for the old cohort (born 1945–1961), the recent cohort (born 1981–1996), and the combined cohort^1^

	Old cohort	Recent cohort	Combined cohort
*n*	31,971	1465	33,436
Weight 8y, kg	26.2 ± 3.5	28.3 ± 5.0^2^	26.3 ± 3.6
Weight 8 y, *z-*score^3^	0.11	0.57	0.13
BMI 8 y, kg/m^2^	15.7 ± 1.4	16.5 ± 2.1^2^	15.8 ± 1.5
BMI 8 y, z*-*score^3^	−0.05	0.41	0.01
Age at PHV, y	14.1 ± 1.1	13.7 ± 1.1^2^	14.0 ± 1.1

1 Values are given as means ± SDs or *n*. PHV, peak height velocity.

2 Significantly different compared with the old cohort tested using a Student *t*-test (*P* < 0.001).

3 Mean weight and mean BMI *z-*scores for use with the CDC growth charts for 8-y-old boys as reference.

**TABLE 2:** Associations for BMI and weight at 8 y of age with age at PHV^1^

	Old cohort (*n* = 31,971)		Recent cohort (*n* = 1465)		Combined cohort (*n* = 33,436)	
	*r^2^* (95% CI)	*P* *^2^*	*r^2^* (95% CI)	*P* *^2^*	*r^2^* (95% CI)	*P* *^2^*
Linear model						
BMI	3.2 (2.8, 3.6)	<0.001	3.3 (1.5, 5.1)	<0.001	3.4 (3.0, 3.8)	<0.001
Weight	5.7 (5.2, 6.2)	<0.001	4.7 (2.6, 6.8)	<0.001	5.9 (5.4, 6.4)	<0.001
BMI + weight	5.8 (5.3, 6.3)	<0.001	4.7 (2.6, 6.8)	<0.001	5.9 (5.4, 6.4)	<0.001
Quadratic model						
BMI	3.5 (3.1, 3.9)	<0.001	3.7 (1.8, 5.6)	0.026	3.6 (3.2, 4.0)	<0.001
Weight	6.2 (5.7, 6.7)	<0.001	5.0 (2.8, 7.2)	0.022	6.3 (5.8, 6.8)	<0.001

1 Associations for BMI and weight at 8 y of age with age at PHV using linear regression in the old cohort (born 1945–1961, *n* = 31,971), the recent cohort (born 1981–1996, *n* = 1465), and the combined cohort (*n* = 33,436). Variances explained (*r^2^*) are percentages. PHV, peak height velocity.

2 *P* values for unadjusted linear correlations are given for the linear models, whereas the *P* values for the quadratic models are the *P* value of the quadratic term to estimate if there is evidence of nonlinearity.

Separate models adjusted for birth year and country of birth, and for the combined cohort, also for the cohort, demonstrated that both body weight and BMI were inversely associated with age at PHV, but the association for body weight was significantly more pronounced than the association for BMI (combined cohort *z*-statistics, *P* < 0.001, **Table 3**). The strength of the association of body weight with age at PHV was mainly unaffected by adjustment for BMI or BMI and BMI^2^ (Table 3), demonstrating that body weight was associated with age at PHV independently of BMI. For the majority of subjects having a body weight ≤40.9 kg (*n* = 33,297), a 1-SD increase in body weight was associated with a 0.25-SD reduction in age at PHV (3.4 mo earlier), whereas no significant association was observed for subjects with a body weight >40.9 kg (**Supplementary Table 1**).

**TABLE 3:** Associations for BMI and weight at 8 y of age with age at PHV.^1^

	Old cohort (*n* = 31,971)	Recent cohort (*n* = 1465)	Combined cohort (*n* = 33,436)
	*β* (95% CI)	*β* (95% CI)	*β* (95% CI)
Separate analyses			
BMI (per SD increase)	−0.18 (−0.19, −0.17)	−0.18 (−0.23, −0.13)	−0.18 (−0.19, −0.17)
Weight (per SD increase)	−0.24 (−0.25, −0.23)	−0.23 (−0.28, −0.18)	−0.24 (−0.25, −0.23)
Adjustments for BMI			
Weight (adjusted for BMI)	−0.27 (−0.29, −0.25)	−0.31 (−0.42, −0.20)	−0.28 (−0.29, −0.26)
Weight (adjusted for BMI and BMI^2^)	−0.28 (−0.29, −0.26)	−0.31 (−0.42, −0.20)	−0.28 (−0.30, −0.26)

1 Associations for BMI and weight at 8 y of age with age at PHV using linear regression in the old cohort (born 1945–1961, *n* = 31,971), the recent cohort (born 1981–1996, *n* = 1465), and the combined cohort (*n* = 33,436). All models are adjusted for birth year and country of birth and, for the combined cohort, also for cohort. All *β* are in SD per SD increase of BMI or weight. The association for weight at 8 y of age with age at PHV was significantly stronger than the association for BMI at 8 y of age with age at PHV when evaluated in the combined cohort (*P* < 0.001 using a *z-*test). PHV, peak height velocity.

To avoid confounding effects of very early puberty on the prepubertal body weight, we performed 2 sensitivity analyses: *1*) Exclusion of individuals with very early age at PHV (before age 11.5 y; **Supplementary Table 2**) and *2*) analyses only using weight and BMI measurements between 6.5 and 8 y of age (**Supplementary Table 3**), yielding very similar results. We also performed a third sensitivity analysis including only boys born in Sweden and with parents born in Sweden, with mainly unchanged results (**Supplementary Table 4**).

## Discussion

In the present longitudinal study using the well-powered BEST Gothenburg cohort, we revisited the “critical weight hypothesis” for regulation of pubertal timing in boys and demonstrated that prepubertal body weight is a more robust inverse predictor of pubertal timing than prepubertal BMI, in boys born both before and during the obesity epidemic.

Recent findings indicate that there is a body weight sensing homeostatic regulation of body weight, the gravitostat, in postpubertal rodents and humans (9–11). In a similar manner, we hypothesized that body weight–sensing regulation of pubertal timing also might occur. If the prepubertal body weight per se modulates pubertal timing, an inverse independent association between prepubertal weight and age at puberty likely exists. Based on a small study already published in *Science* in 1970, Frisch et al. proposed the critical body weight hypothesis, stating that to initiate puberty, girls need to reach a critical level of body weight and thereby a certain fat mass (12, 13). However, subsequent association studies have focused on the inverse association between BMI and pubertal timing observed in most but not all studies (12, 15, 19, 22, 23, 25, 26, 29, 30). A longitudinal study in girls (18), a cross-sectional study in boys (8), and 2 small longitudinal studies in boys (32, 33) have indicated that both body weight and BMI are inversely associated with pubertal timing, but no previous longitudinal well-powered study has compared the strengths of the associations for prepubertal weight and BMI with pubertal timing in boys. We identified a nonlinear association between body weight and age at PHV. An inverse association between body weight and age at PHV was observed below, but not above, a body weight of 40.9 kg. It is possible that the small part of the sample with a body weight >40.9 kg resulted in insufficient power to identify an association. Alternatively, the body weight required to initiate puberty is reached at 40.9 kg, and there is no additional impact of body weight on pubertal onset for boys with body weight >40.9 kg. Of note, the present finding of a robust BMI-independent inverse association between prepubertal weight and age at puberty in boys supports the critical body weight hypothesis.

Initiation of puberty most likely involves an activation of the GnRH pulse generator, but the underlying mechanism is still unclear (6–8). The present finding of an inverse robust association between prepubertal weight and age at puberty indicates that body weight is of importance for timing of the pubertal growth spurt in boys and supports the concept that body weight sensing per se initiates an afferent signal for modulation of the GnRH pulse generator in a similar manner as previously described for the regulation of body weight in postpubertal rodents and humans (9–11). Alternative hypotheses have been that fat-derived leptin (31) or maturational processes within the central nervous system modulate the activity of the GnRH pulse generator and, thereby, the timing of puberty (13).

A strength of the present study was that sensitivity analyses, excluding individuals with very early age at PHV (before age 11.5 y) or using weight measurements only between 6.5 and 8 y of age, to avoid confounding effects of very early puberty, yielded very similar results. Furthermore, the BEST cohort is the first large population-based cohort of males to provide information on childhood prepubertal weight and BMI, as well as the opportunity for objective assessment of age at pubertal timing that captures the entire pubertal period. The strengths of the present study also include the fact that similar results were observed for the cohort before (old cohort) and during (recent cohort) the obesity epidemic, suggesting that the inverse association between prepubertal weight and age at puberty is not only caused by the obesity epidemic. A major limitation with the present study is that we did not have data for girls. Another limitation of this observational study is that we cannot prove causality and, therefore, it is possible that both childhood body weight and pubertal timing are coregulated but have no causal relationship to each other. Lastly, we do not have information on confounders such as exposure to endocrine disrupting chemicals or hormonal disorders.

In conclusion, prepubertal body weight is a more robust inverse predictor of pubertal timing than prepubertal BMI in boys. We propose that body weight sensing constitutes a feedback mechanism to regulate pubertal timing.

## Supplementary Material

nqaa304_Supplemental_File

## Notes

Supported by grants from the Swedish Research Council and the Swedish Government (under the Agreement for Medical Education and Research), the Lundberg Foundation, the Torsten Söderberg Foundation, the Novo Nordisk Foundation, the Knut and Alice Wallenberg Foundation, and the Heart and Lung Foundation. The funding sources of the study had no role in the study design, implementation, analysis, and interpretation of the data.

Supplementary Tables 1–4 and Supplementary Figure 1 are available from the “Supplementary data” link in the online posting of the article and from the same link in the online table of contents at https://academic.oup.com/ajcn/.

Data Availability: Data described in the manuscript, code book, and analytic code will not be made available because of privacy and ethical restrictions. However, anonymized data that are minimally required to reproduce results can be made available from the corresponding author upon reasonable request, upon approval from the University of Gothenburg, if the data can be made available according to mandatory national law.

Abbreviations used: BEST, BMI Epidemiology Study; GnRH, gonadotropin-releasing hormone; PHV, peak height velocity; PIN, personal identity number.
