# Supplementary material for: Revisiting the critical weight hypothesis for regulation of pubertal timing in boys
Source: Am J Clin Nutr. 2020 Nov 12;113(1):123–8. doi: 10.1093/ajcn/nqaa304 (PMC7779230; doi:10.1093/ajcn/nqaa304)
Supplement: nqaa304_Supplemental_File [file nqaa304_supplemental_file.docx]

**Revisiting the critical weight hypothesis for regulation of pubertal timing in boys, Maria Bygdell, Online supplementary material**

**Online supplementary material**

**Revisiting the critical weight hypothesis for regulation of pubertal timing in boys**

Maria Bygdell, Jenny M Kindblom, John-Olov Jansson, Claes Ohlsson

**Supplementary Tables**

**Supplementary Table 1. Associations for weight at 8 years of age with age at PHV stratified according to a weight below or above 40.9 kg at 8 years of age.**

|  | **Combined cohort**  (n=33,436) |
| --- | --- |
|  | *Beta (95%CI)* |
| **≤40.9 kg** (n=33,297) |  |
| Weight (per SD increase) | -0.25 (-0.27, -0.24) |
|  |  |
| **>40.9 kg** (n=139) |  |
| Weight (per SD increase) | -0.05 (-0.24, 0.14) |

Associations for weight at 8 years of age with age at PHV in the combined cohort (n=33,436) stratified according to weight at 8 years of age below or above 40.9 kg. All models are adjusted for birth year, country of birth, and cohort. Betas are in SD per SD increase of weight. PHV=Peak Height Velocity, CI=Confidence Interval, SD=Standard Deviation.

**Supplementary Table 2. Associations for BMI and weight at 6.5-8 years of age with age at PHV.**

|  | **Old cohort**  (n=31,408) | **Recent cohort**  (n=1,261) | **Combined cohort**  (n=32,669) |
| --- | --- | --- | --- |
|  | *Beta (95%CI)* | *Beta (95%CI)* | *Beta (95%CI)* |
| **Separate analyses** |  |  |  |
| BMI (per SD increase) | -0.16 (-0.17, -0.15) | -0.16 (-0.22, -0.11) | -0.16 (-0.17, -0.15) |
| Weight (per SD increase) | -0.24 (-0.25, -0.22) | -0.21 (-0.27, -0.16) | -0.24 (-0.25, -0.22) |
|  |  |  |  |
| **Adjustments for BMI** |  |  |  |
| Weight (Adjusted for BMI) | -0.29 (-0.31, -0.27) | -0.32 (-0.44, -0.20) | -0.29 (-0.31, -0.28) |
| Weight (Adjusted for BMI and BMI^2^) | -0.30 (-0.31, -0.28) | -0.31 (-0.43, -0.19) | -0.30 (-0.32, -0.28) |

Associations for BMI and weight at 6.5-8 years of age with age at PHV using linear regression in the old cohort (born 1945-1961, n=31,408), the recent cohort (born 1981-1996, n=1,261), and the combined cohort (n=32,669). All models are adjusted for birth year, age at measurement, and country of birth and for the combined cohort also for cohort. Betas are in SD per SD increase of BMI or weight. BMI=Body Mass Index, PHV=Peak Height Velocity, CI=Confidence Interval, SD=Standard Deviation.

The association for weight at 6.5-8 years of age vs age at PHV was significantly stronger than the association for BMI at 6.5-8 years of age vs age at PHV when evaluated in the combined cohort (p <0.001 using a Z test).

**Supplementary Table 3. Associations for BMI and weight at 8 years of age with age at PHV excluding individuals with age at PHV below 11.5 years of age.**

|  | **Old cohort**  (n=31,296) | **Recent cohort**  (n=1,426) | **Combined cohort**  (n=32,722) |
| --- | --- | --- | --- |
|  | *Beta (95%CI)* | *Beta (95%CI)* | *Beta (95%CI)* |
| **Separate analyses** |  |  |  |
| BMI (per SD increase) | -0.16 (-0.17, -0.15) | -0.17 (-0.23, -0.12) | -0.16 (-0.17, -0.15) |
| Weight (per SD increase) | -0.22 (-0.24, -0.21) | -0.21 (-0.26, -0.16) | -0.22 (-0.23, -0.21) |
|  |  |  |  |
| **Adjustments for BMI** |  |  |  |
| Weight (Adjusted for BMI) | -0.27 (-0.29, -0.25) | -0.29 (-0.40, -0.17) | -0.27 (-0.29, -0.25) |
| Weight (Adjusted for BMI and BMI^2^) | -0.27 (-0.29, -0.26) | -0.29 (-0.40, -0.17) | -0.28 (-0.30, -0.26) |

Associations for BMI and weight at 8 years of age with age at PHV using linear regression in the old cohort (born 1945-1961, n=31,296), the recent cohort (born 1981-1996, n=1,426), and the combined cohort (n=32,722), excluding individuals with age at PHV below 11.5 years of age. All models are adjusted for birth year, and country of birth and for the combined cohort also for cohort. Betas are in SD per SD increase of BMI or weight. BMI=Body Mass Index, PHV=Peak Height Velocity, CI=Confidence Interval, SD=Standard Deviation.

The association for weight at 8 years of age vs age at PHV was significantly stronger than the association for BMI at 8 years of age vs age at PHV when evaluated in the combined cohort (p < 0.001 using a Z test).

**Supplementary Table 4. Associations for BMI and weight at 8 years of age with age at PHV for individuals born in Sweden and with parents born in Sweden.**

|  | **Old cohort**  (n=26,749) | **Recent cohort**  (n=970) | **Combined cohort**  (n=27,719) |
| --- | --- | --- | --- |
|  | *Beta (95%CI)* | *Beta (95%CI)* | *Beta (95%CI)* |
| **Separate analyses** |  |  |  |
| BMI (per SD increase) | -0.18 (-0.19, -0.17) | -0.22 (-0.28, -0.16) | -0.18 (-0.19, -0.17) |
| Weight (per SD increase) | -0.24 (-0.25, -0.23) | -0.26 (-0.32, -0.20) | -0.24 (-0.25, -0.23) |
|  |  |  |  |
| **Adjustments for BMI** |  |  |  |
| Weight (Adjusted for BMI) | -0.27 (-0.29, -0.25) | -0.33 (-0.46, -0.19) | -0.28 (-0.30, -0.26) |
| Weight (Adjusted for BMI and BMI^2^) | -0.28 (-0.30, -0.26) | -0.32 (-0.46, -0.18) | -0.28 (-0.30, -0.26) |

Associations for BMI and weight at 8 years of age with age at PHV using linear regression in the old cohort (born 1945-1961, n=26,749), the recent cohort (born 1981-1996, n=970), and the combined cohort (n=27,719). All models are adjusted for birth year, and for the combined cohort also for cohort. Betas are in SD per SD increase of BMI or weight. BMI=Body Mass Index, PHV=Peak Height Velocity, CI=Confidence Interval, SD=Standard Deviation.

The association for weight at 8 years of age vs age at PHV was significantly stronger than the association for BMI at 8 years of age vs age at PHV when evaluated in the combined cohort (p < 0.001 using a Z test).

**Supplementary Figures**

**Supplementary Figure 1.** Flow chart of included participants in (a) the old cohort and (b) the recent cohort. BMI=Body Mass Index, PHV=Peak Height Velocity, PIN=Personal Identity Number

**Supplementary Figure 1A**

**Supplementary Figure 1B**
